# Effect of a Counseling Session Bolstered by Text Messaging on Self-Selected Health Behaviors in College Students: A Preliminary Randomized Controlled Trial

**DOI:** 10.2196/mhealth.6638

**Published:** 2017-05-17

**Authors:** Janice Sandrick, Doreen Tracy, Arn Eliasson, Ashley Roth, Jeffrey Bartel, Melanie Simko, Tracy Bowman, Karen Harouse-Bell, Mariam Kashani, Marina Vernalis

**Affiliations:** ^1^ Coordinated Program in Nutrition and Dietetics Seton Hill University Greensburg, PA United States; ^2^ Integrative Cardiac Health Project Department of Medicine Walter Reed Military Medical Center Bethesda, MD United States; ^3^ Psychology Program Seton Hill University Greensburg, PA United States

**Keywords:** health behaviors, diet habits, exercise, sleep, text telecommunications, universities

## Abstract

**Background:**

The college experience is often the first time when young adults live independently and make their own lifestyle choices. These choices affect dietary behaviors, exercise habits, techniques to deal with stress, and decisions on sleep time, all of which direct the trajectory of future health. There is a need for effective strategies that will encourage healthy lifestyle choices in young adults attending college.

**Objective:**

This preliminary randomized controlled trial tested the effect of coaching and text messages (short message service, SMS) on self-selected health behaviors in the domains of diet, exercise, stress, and sleep. A second analysis measured the ripple effect of the intervention on health behaviors not specifically selected as a goal by participants.

**Methods:**

Full-time students aged 18-30 years were recruited by word of mouth and campuswide advertisements (flyers, posters, mailings, university website) at a small university in western Pennsylvania from January to May 2015. Exclusions included pregnancy, eating disorders, chronic medical diagnoses, and prescription medications other than birth control. Of 60 participants, 30 were randomized to receive a single face-to-face meeting with a health coach to review results of behavioral questionnaires and to set a health behavior goal for the 8-week study period. The face-to-face meeting was followed by SMS text messages designed to encourage achievement of the behavioral goal. A total of 30 control subjects underwent the same health and behavioral assessments at intake and program end but did not receive coaching or SMS text messages.

**Results:**

The texting app showed that 87.31% (2187/2505) of messages were viewed by intervention participants. Furthermore, 28 of the 30 intervention participants and all 30 control participants provided outcome data. Among intervention participants, 22 of 30 (73%) showed improvement in health behavior goal attainment, with the whole group (n=30) showing a mean improvement of 88% (95% CI 39-136). Mean improvement in any behavioral domains was not seen in the control group. Intervention participants also increased their exercise significantly compared with controls, regardless of their self-selected goal category. The increased exercise was paralleled by significantly lower fasting glucose levels.

**Conclusions:**

The health coaching session plus tailored SMS text messages improved self-selected health behaviors with a modest ripple effect to include unselected health behaviors.

**Trial Registration:**

Clinicaltrials.gov NCT02476604; https://clinicaltrials.gov/ct2/show/NCT02476604 (Archived by WebCite at http://www.webcitation.org/6qAAryS5t)

## Introduction

During the transition to campus life, college students face challenges related to eating behaviors, physical activity, stress, and sleep [1-4]. Numerous factors may contribute to unhealthy lifestyles among students [5]. The all-you-can-eat dining facilities, time constraints, and lack of food preparation facilities in the dormitories affect food choices [5]. Diminished physical activity [6] and heightened levels of perceived stress [2], especially in women [7], can influence weight. It has been well documented that college students experience significant weight gain and changes in body composition, notably in their freshman year, putting them at higher risk for chronic diseases [8-10]. A need exists for effective strategies to encourage college students to adopt and maintain healthy lifestyles.

Mobile technology is commonplace today, particularly on college campuses [11]. As defined by a recent review, mobile technology broadly includes bidirectional message-type activities via conventional mobile phones, smartphones, and portable laptop computers that allow for exchange of text messages (short message service, SMS), electronic mail, live video feeds, and Internet access [11]. Because nearly all students have cell phones with them most of their waking time [11], text message–based interventions represent a promising opportunity to offer remote health coaching to students on the go [12,13]. Since the introduction of mobile phone–based interventions to promote behavior change, numerous researchers have reported positive outcomes [13]. For example, research has investigated the efficacy of mobile phone–based interventions on smoking cessation [14-17], weight loss [18-21], physical activity [22-25], and improving clinical outcomes of pregnancy [26-28]. A mobile phone–based intervention that encourages healthy changes in a variety of lifestyle behaviors would have clear advantages, offering efficiency as several health behaviors could be addressed through a single app. However, most mobile phone–based interventions have focused on a single health objective to date.

This preliminary randomized controlled trial aimed to evaluate the effect of a single coaching session bolstered by tailored text messages on self-selected health goals (described in further detail in the Methods section) in a sample of college students. We hypothesized that the coaching plus text message–based intervention would result in students successfully attaining their specified goals in the intervention group, which was randomly selected from interested participants. In this study, we also performed a comparison with a control group to determine whether or not the goal-oriented improvements would be associated with improvements in other lifestyle behaviors to benefit the students’ health in areas beyond their self-selected area of focus. The study also incorporated objective tests (serum glucose and cholesterol) that may be responsive to improved lifestyle behaviors.

## Methods

### Participants

This study was conducted at a small university in western Pennsylvania from January 2015 to May 2015 in collaboration with the Integrative Cardiac Health Project, Walter Reed Military Medical Center in Bethesda, Maryland. The university Institutional Review Board and the Human Research Protection Office of the US Army Medical Research and Materiel Command approved the study protocol for human subject research. The study protocol was registered on ClinicalTrials.gov with trial number 375278-3 (ClinicalTrials.gov Identifier: NCT02476604). Written informed consent was obtained from eligible participants before enrolment. The full trial protocol may be obtained by written request to the corresponding author.

Participants were recruited using various strategies such as classroom announcements, posted advertisements, flyers, and booths located in student common areas on the university campus. Students were eligible to participate if they were full-time students at the university and did not have any exclusion criteria. The exclusion criteria included (1) age less than 18 years and greater than 30 years; (2) pregnancy, suspected pregnancy, or plans to become pregnant; (3) diagnosis of a chronic medical condition; (4) history of an eating disorder; and (5) taking any prescribed medication other than an oral contraceptive.

### Data Collection

A comprehensive health evaluation was performed by certified health coaches at enrolment in the study. Health coach certification was conferred by the American Council on Exercise, an accredited body that teaches skills for active listening and providing motivational feedback. The coaching skill set is applicable for a variety of health topics including diet, exercise, stress management, and sleep habits. The comprehensive evaluation upon enrolment in the study included a focused physical assessment and collection of information such as past medical history, current medications, and a review of systems using typical demographic forms. At baseline and following the 8-week intervention period, participants completed validated behavioral surveys (described in detail below) assessing dietary habits, exercise activity, levels of perceived stress, and sleep practices. Blood testing for fasting glucose and cholesterol was also performed before and after the intervention (details below).

### Behavioral Surveys

(1) Rate Your Plate [29] (RYP) dietary assessment: The RYP consists of 27 questions with responses from 1 to 3 yielding a scoring range from 27 to 81. A higher RYP score indicates a more healthful eating pattern. Convergent validity for the RYP was validated in another setting in a comparison study with a previously validated survey tool, the Willett food frequency questionnaire [30]. Using the Pearson product-moment correlation, the two questionnaires showed a strong degree of correlation with *r*=.4-.5 (*P*<.001), on a range of specific dietary variables [29].

(2) International Physical Activity Questionnaire [31] (IPAQ): The IPAQ asks respondents to recall their physical activity levels for a “usual week” and relates the recalled activities to metabolic equivalents (METs) for standardization of data. The IPAQ has been validated across 12 nations as an exercise survey questionnaire including college-aged adults to be self-administered or administered by interview. The IPAQ provides reproducible data (test-retest reliability, concurrent validity, and criterion IPAQ validity against accelerometry) with a Spearman rho of .8 [31].

(3) Perceived Stress Scale [32] (PSS 14): This 14-item questionnaire asks the respondent how often certain experiences of stress occurred in the last month and is designed to measure the degree to which situations in one’s life are appraised as stressful. With item responses from 0 to 4, the range of possible scores is 0 to 56, with higher scores correlating with higher stress. The PSS is designed for use in community samples with at least a junior high school education. The items are easy to understand and the response alternatives are simple to grasp. Moreover, the questions are quite general in nature and hence relatively free of content specific to any subpopulation group. Scores in the low 20s reveal modest stress levels, whereas scores approaching 30 are substantial and concerning. A validation study [32] shows that the PSS 14 has an internal consistency reliability of .85 by Cronbach alpha and a test-retest reliability of .85.

(4) Pittsburgh Sleep Quality Index [33] (PSQI): The PSQI is a self-rated questionnaire that assesses sleep quality and disturbances over a 1-month time interval. Nineteen individual items generate 7 component scores whose sum yields a global score with a range of 0 to 21. A global score of greater than 5 indicates a poor sleeper. Sleep perturbations can be categorized by scores: 0 to 5 is a good sleep score; 6 to 10 shows mild sleep difficulty; 11 to 15 moderate sleep difficulty; and 16 to 21 severe sleep difficulty. The psychometric and clinical properties of the PSQI suggest its utility both in clinical practice and research activities. A PSQI greater than 5 has a diagnostic sensitivity of 90% and specificity of 87% (kappa > .75, *P*<.001) [33].

(5) Epworth Sleepiness Scale [34] (ESS): The ESS is the most widely used tool to estimate the subjective symptom of daytime sleepiness. Respondents are asked to use a scale of 0 to 3 to estimate their likelihood of dozing in 8 different situations in recent weeks. The individual scores are summed and possible scores range from 0 to 24. A score greater than 10 indicates excessive daytime sleepiness. The ESS has a sensitivity of 94% and a specificity of 100% for detecting excessive daytime sleepiness [34].

(6) Visual Analog Fatigue Scale [35] (FS): The FS was developed by the Stanford Patient Education Research Center. The scale was tested with 122 individuals, with a mean value of 4.9 and standard deviation of 2.7. This fatigue scale asks respondents to express their experience of fatigue from 0 to 10 for the previous 2-week period. Individuals denoting scores of 0 to 4 have no-to-minimal fatigue, those who circle 5 to 7 express milder fatigue, and those selecting 8 to 10 experience more severe fatigue.

### Blood Collection

A certified phlebotomist collected two blood samples from each participant, one sample upon enrolment in the study and the second sample at the end of the 8-week study period. Participants in both the intervention arm and the control arm received a US $50.00 payment for each of the two blood samples taken. Participants were asked to fast for 12 hours before blood collection. All blood collections occurred between 7:00 AM and 9:00 AM. Serum samples were collected in vacutainers and separated by centrifuging at 4150 rpm for 5 minutes using a Sorvall Legend Mach 1.6 centrifuge (Thermo Fisher Scientific Inc). Samples were stored at 22°C and analyzed for total cholesterol and glucose at a regional commercial laboratory.

### Intervention

The participants were randomly assigned in a one-to-one ratio by a computer program to one of two groups for the duration of the study. As part of the informed consent procedure, participants were made aware that the study involved a flip-of-the-coin chance that they may experience different components of the study compared with another participant. After randomization, participants were further informed regarding the particulars of their expected experience. One group received the intervention of behavioral goal setting with a health coach (certified by the American Council on Exercise) followed by text messaging throughout the 8-week study period (described in detail below). The other group served as controls, receiving their baseline survey results and blood test results in writing but not setting behavioral goals with the health coach and not receiving text messages during the study period.

**Table 1:** Examples of behavioral goals with intervention messages.

Goals and messages		Nutrition	Exercise	Stress	Sleep
**Goal**		Increase fruit and vegetable intake from 3 per day to 5 per day.	Increase exercise from 0 to 3 times per week for 20 minutes. (60 minutes per week)	Make a “to-do” list 5 days per week instead of 0 days per week.	Make bedtime 11:00 PM instead of midnight.
**Example messages**					
	Action-oriented	Try to add two servings of fruit to your day, a handful of grapes, a crisp apple, etc. Natural fruit is a great source of fiber, provides fullness and sweetness.	Try this workout: 5 minutes of a light jog, 1 minute of sprinting followed by 1 minute of low speed jogging, repeat 5 times. Jog for 5 minutes as your cool down.	Try to organize for 15 minutes each day. This could mean anything from sorting mail to throwing out mystery foods in the refrigerator. Just 15 minutes per day can make a huge difference over time! Enjoy your weekend!	Did you know that stretching before bed could help you sleep better? Give this stretch a try: Sit on floor with pillow in front of you. Bend left knee, bringing bottom of left foot to right inner thigh. Lift butt and extend right leg behind you. Stay centered and lean forward from hips, placing head on pillow. Extend arms forward, elbows are slightly bent. Hold for 8-10 breaths. Roll back up. Switch legs; repeat.
	Motivational	Good job with your fruits and veggies! Looks like you’ve improved!	No matter how slow you go. You are still lapping everyone on the couch.	Make each day count. We don’t have all the time in the world. So focus on today and do the things you really want to do. :)	Perform at your highest potential. Sleep prepares your body and mind for the next day’s endeavors.
	Informational	Too much salt in our diet can raise blood pressure. Most fruits and vegetables are naturally low in salt and can help improve blood pressure. Focus on putting more fresh food like fruits and vegetables into your meals.	Exercise releases endorphins, which creates feelings of happiness and euphoria.	I heard the app “Simplenotes” is a good one for your iPhone for organization and it’s free...you should look into it!	Did you know? Watching television before bedtime can actually stimulate the mind rather than relax it. Listening to audiobooks or music may be a better choice.

Following baseline data collection, results were aggregated so that study participants could compare their results to normative standards. This allowed participants to visualize potential areas of their health behaviors that would benefit from improvement. Participants were sent their health assessment to review in preparation for a meeting in person with the health coach. During the face-to-face meeting with the health coach, participants in the intervention group were asked to set one behavioral goal in a domain of diet, exercise, stress management, or sleep. The behavioral goal represented an actionable behavior that the participant could work to improve. (See Table 1 for sample goals.) Weight loss was not included as a goal because weight loss itself is a result of behavioral change, not a behavior itself. Health coaches also used the face-to-face interaction to learn about the participant’s routine, habits, and motivational cues providing relevant information for tailoring messages. This initial face-to-face meeting required 45 minutes to 1 hour to complete.

### SMS Text Messages

Before the creation of text message content, focus groups were conducted in order to understand preferences of the college student target population (A Roth, unpublished data, September, 2014). The dialogue captured during these focus groups suggested that college students preferred a mix of action-oriented, informational, and motivational messages that were succinct and sent no more than three times per week (Table 1). The investigators elected not to exceed the frequency of 3 messages per week so as to avoid turning an encouraging communication into an aggravation that would be quickly deleted. The health coaches disseminated intervention SMS text messages through a custom iOS application designed by programmers working with the Integrative Cardiac Health Project. Study participants downloaded the iOS application to a university-issued iPad or a personal iPhone. Intervention messages were delivered on a regular schedule on Tuesdays and Thursdays at 9:00 AM and Saturdays at 11:00 AM for the study period of 8 weeks. The schedule for delivery of messages was chosen to distribute the messages across the week at times that would not be disruptive of the students’ living schedule. Students retained the freedom to check for messages at their convenience. There were two health coaches hired to conduct the study. Both of the coaches worked in equal measure with participants who were randomized to the intervention arm and with those who were randomized to the control arm. Once a participant was assigned to one of the health coaches, that participant received all contact from that particular health coach for the duration of the study. Health coaches were in contact with the participants randomized to the control arm at the beginning of the study and again at study completion. The health coaches did not have contact with the control participants during the 8 weeks of the study period. Both coaches were certified for training in the same health coaching techniques and provided text messages to the participants randomized to the intervention arm using guidance from the participants themselves as to what sort of messages would help them stay on track to reach their self-selected health behavior goal. Participants in the intervention arm were asked weekly via the application to complete brief behavioral assessments (BA). The BA included a question addressing progress toward the self-selected goal. The coaches used BA feedback to customize text messages to address barriers impeding goal attainment. Some participants asked for more motivational type of text messages, whereas other participants opted for action-oriented messages that instructed them on their behaviors. A third type of message preferred by some participants was an informational-type text message. (See Table 1 for sample messages.) The coaches required approximately one hour for each intervention participant per week to read the brief BA feedback messages and to customize the text messages to be disseminated to each participant. The self-selected behavioral goal served as a basis for text messages designed to encourage goal attainment. Half of the messages sent as part of the intervention were directed toward the self-selected behavioral goal and half of the messages targeted other health behaviors more generally. Supervision of the text message development was provided by the study’s principal investigator.

### Sample Size Estimation

The RYP dietary assessment tool served as an outcome measure to determine the sample size estimation because the investigators had a past experience with the RYP tool and could estimate an effect size of the intervention on this parameter. It was assumed that similar responsiveness in the other outcome measures would be seen and that by using a general “improvement score” for each behavioral dimension, participants could be offered the freedom to choose any of the four behaviors for concentration of their improvement efforts. Dietary changes in RYP were expected to average 10 (SD 2) points for the intervention group compared with zero change (same standard deviation) for the control group. With a power of 90% for rejecting the null hypothesis and using a one-sided test with alpha = 2.5% (as we expected an increase in dietary adherence and minimal chance of a decrease in adherence), the sample size was found to be 25 participants in each arm of the study. Accounting for a 30% dropout rate, a total of 70 participants would be recruited, 35 participants for each arm.

### Statistical Methods

Statistical analysis was performed using SPSS for Macintosh version 22.0.0.0 (IBM, 2013). Descriptive statistics were calculated for variables including means, standard deviations, medians, and minimum and maximum values. Skewness and kurtosis values of the dependent variables were analyzed for significant deviation from a normal distribution. Further comparisons between participants randomized to the intervention group and those serving as controls were made using two-sample *t* tests or chi-square tests as appropriate, with a *P* value <.05 indicating statistical significance.

Because participants set unique behavioral goals, we used a standardized indicator that operationalized “success” as the percentage of each participant’s goal that was attained at the conclusion of the study. For example, at intake one participant completed 7758 steps per day. She set a goal of 10,000 steps per day, and by the completion of the study, she had completed 9008 steps per day. Thus, she attained 56% (ie, 100 × [9008−7758]/[10,000−7758]) of her goal. The intervention would be considered successful if the mean percentage of goal attainment was statistically significantly greater than 0.

## Results

### General

**Figure 1:**
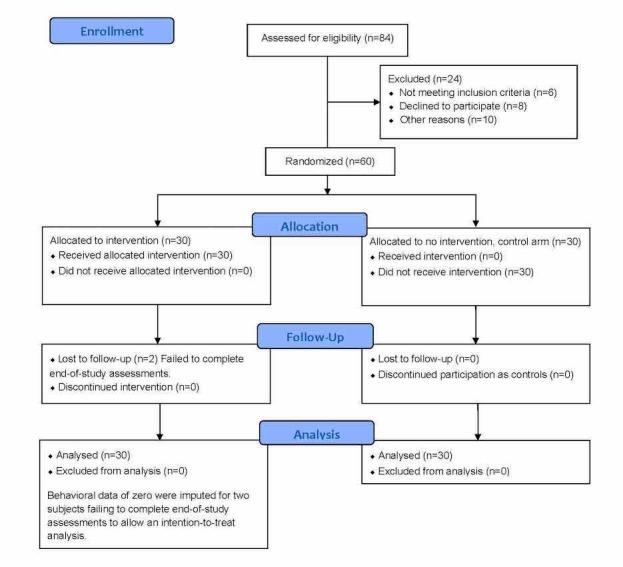
CONSORT flow diagram.

A total of 84 potential participants were screened for eligibility to participate in the study. Following baseline data collection, 3 participants were released due to prescription medication usage and 1 because baseline blood pressure results showed hypertension deserving medical attention. Other attrition factors included withdrawing from the university, change in student status, electing not to participate in the study due to time constraints, or other personal reasons (Figure 1). Of 60 enrolled participants, 30 were randomized to the intervention arm and 30 to the control arm. For 2 participants in the intervention arm, end-of-study data were not obtained to determine success or failure of the intervention with respect to goal attainment. In order to maintain an intention-to-treat analysis, these 2 participants were assigned zero progress toward their behavioral goals and their data were retained in the final analysis.

Demographic information on the 60 participants is presented in Table 2. The study population comprised young adults, predominantly white (87%), and the majority were women (68%). A majority of participants were freshmen (43%) or sophomores (30%), and most participants (80%) lived on campus during the study period. As shown in Table 2, there were no differences in demographic characteristics between participants randomized to the intervention and control arms.

**Table 2:** Demographic Information.

Demographic characteristics	All participants (n=60)	Control (n=30)	Intervention (n=30)	*P* value^a^
Age in years, mean (SD)	19.4 (1.0)	19.3 (0.9)	19.5 (1.1)	.38
Gender (women), n (%)	41( 68)	20 (67)	21 (70)	.79
Race^b^	52W, 5B, 2H, 1A	24W, 4B, 1H, 1A	28W, 1B, 1H	.38
Grade point average, mean (SD)	3.5 (0.4)	3.5 (0.4)	3.6 (0.3)	.16
Year of study^c^	26F, 18So, 9J, 7Sr	12F, 11So, 5J, 2Sr	14F, 7So, 4J, 5Sr	.49
On/off campus residence	48/12	24/6	24/6	>.99
Active in varsity sport, n (%)	27 (45)	16 (53)	11 (37)	.42

^a^*P* value signifies level of statistically significant differences by *t* test between control and intervention subjects.

^b^W=white, B=black, H=Hispanic, A=Asian.

^c^F=freshman, So = sophomore, J=junior, Sr = senior.

### Goal Attainment for Intervention Participants

The computer server was queried to compile information on messaging. During the course of the study, 1160 messages were sent to intervention participants and 982 messages (85%) were viewed.

Values for goal attainment were acceptable for a normal distribution (D_27_=.95, *P*=.19; skewness=0.28, SE=0.45; and kurtosis=1.18, SE=0.87). Of the 30 participants who received the intervention, 22 (73%) showed improvements in their self-declared behavioral goal, 6 of 30 (20%) did not make gains toward their behavioral goal, and 2 (7%) did not provide data that could be evaluated. For analysis, the 2 participants who failed to complete end-of-study assessments were assigned zero values for behavioral goal attainment and were therefore considered failures of the intervention. For the entire intervention group (n=30), the success rate of 88% was calculated by averaging the percentages of each participant’s goal (positive gains, negative, and zeros) that was attained at the conclusion of the study. For the 6 participants who did not make gains toward their goals, 1 reported no change and 5 reported negative movement, that is, movement away from their goals, averaging negative 66%. A one-sample *t* test on participants’ percent of goal attainment scores was used to determine whether or not their mean was significantly greater than 0 (a score of 0 or less would indicate that the intervention was not effective because the participant would have achieved 0% of his or her goal or, indeed, had regressed on the goal indicator following the intervention). The one-sample *t* test indicated that the percent of goal attainment of 88% (for n=30) was significantly greater than 0 (*t*_26_=3.68, *P*<.001, Cohen d=0.71, 95% CI [38.56-136.11]). Our hypothesis that the coaching plus text intervention would help participants’ goal attainment was supported by the data.

Additionally, data were examined to determine whether or not the participants’ success depended upon the category of their self-selected behavioral goal. Of the 30 participants in the intervention group, 10 selected dietary goals, 8 selected exercise goals, whereas there were 6 participants each who selected goals in stress management and in sleep. Based upon the category of behavioral goal, there were no differences in means for percentage of goal attainment (*F*_3,20_=.16, *P*=.92, partial eta-squared = .29). The breakdown in the percentages of goal attainment across the 4 categories was diet (131%), exercise (75%), stress management (69%), and sleep (98%).

Since the control participants did not establish behavioral goals, it was not possible to measure control group progress toward a composite behavioral goal at the outcome of the study as was done for the intervention participants. However, the validated behavioral surveys were completed by control participants at baseline and at the outcome date of the study. As was anticipated, control participants did not show statistically significant changes in any of the behavioral domains (for diet *P*=.55, for exercise *P*=.77, for stress management *P*=.75, and for sleep *P*=.93).

### Broader Effects of a Self-Selected Behavior Goal

Upon comparing intervention participants with the control group, irrespective of their particular behavioral goals, it was found that exercise was the only behavior difference, with intervention participants increasing their reported exercise more than controls to a statistically significant degree (Table 3). Intervention participants increased their exercise by 19% compared with control participants, who decreased their exercise by 6%. There were no other differences in health behaviors or symptoms between intervention participants and controls, implying that there was only a modest “ripple effect” to produce behavioral improvements outside of the self-selected behavioral goal.

Participants receiving the intervention showed a statistically significant improvement in fasting blood glucose compared with controls (6% decrease vs 1% decrease), while cholesterol levels were not different (Table 3).

**Table 3:** Lifestyle behaviors, symptoms, and laboratory data at program baseline and program completion.

Behavior or Objective Measure	Program baseline				Program completion			
	All participants (n=60)	Control (n=30)	Intervention (n=30)	*P* value^a^	All participants (n=60)	Control (n=30)	Intervention (n=30)	*P* value^a^
Diet (RYP)^b^	61.4 (SD 7.8)	61.5 (SD 7.7)	61.3 (SD 8.1)	.91	63.0 (SD 8.9)	62.7 (SD 8.3)	63.6 (SD 9.6)	.81
Exercise (IPAQ)^c^	2416 (SD 2508)	2208 (SD 2106)	2632 (SD 2889)	.52	2590 (SD 1965)	2074 (SD 1410)	3144 (SD 2324)	.04
Stress (PSS-14)^d^	23.1 (SD 8.0)	23.1 (SD 8.9)	23.0 (SD 7.0)	.99	23.9 (SD 8.7)	23.8 (SD 9.3)	24.0 (SD 8.1)	.94
Sleep (PSQI)^e^	5.2 (SD 2.7)	5.3 (SD 2.3)	5.1 (SD 3.0)	.81	5.5 (SD 3.0)	5.9 (SD 3.2)	5.2 (SD 2.9)	.37
Sleepiness (ESS)^f^	7.8 (SD 3.8)	8.1 (SD 4.3)	7.5 (SD 3.2)	.55	8.5 (SD 4.0)	8.8 (SD 3.8)	8.3 (SD 4.2)	.63
Fatigue (SFS)^g^	4.1 (SD 2.0)	3.7 (SD 2.2)	4.4 (SD 1.9)	.21	4.6 (SD 2.0)	4.3 (SD 2.0)	4.9 (SD 2.0)	.25
Fasting glucose^h^	78.8 (SD 6.4)	79.0 (SD 6.7)	78.6 (SD 6.3)	.83	76.1 (SD 8.4)	78.2 (SD 8.2)	74.0 (SD 8.3)	.05
Fasting cholesterol^h^	159.4 (SD 30.1)	157.1 (SD 28.5)	161.7 (SD 32.0)	.56	159.7 (SD 29.1)	160.5 (SD 29.3)	158.9 (SD 29.4)	.83

^a^*P* value signifies level of statistically significant differences by *t* test between control and intervention subjects.

^b^RYP: Rate Your Plate Dietary Questionnaire; of the 81 total possible points, higher score indicates a healthier diet.

^c^IPAQ: International Physical Activity Questionnaire in metabolic equivalent-minutes per week, higher score equates with greater activity level.

^d^PSS-14: Perceived Stress Scale, of 56 total possible points, higher score indicates greater level of perceived stress.

^e^PSQI: Pittsburgh Sleep Quality Index, of 21 possible points, lower score indicates better sleep quality.

^f^ESS: Epworth Sleepiness Scale, of 24 possible points, higher score indicates greater daytime sleepiness.

^g^SFS: Stanford Fatigue Scale, of 10 possible points, higher score indicates greater severity of fatigue symptom.

^h^Fasting glucose and fasting cholesterol in mg/dL.

## Discussion

### Principal Findings

The main research hypothesis of this study was that a single counseling session followed by a text message–based intervention would result in the attainment of a self-selected health behavior goal. A secondary analysis was performed to determine whether or not the intervention directed at a particular behavior might also have a broader effect by encouraging other healthful behavior changes outside of the behavior goal of choice.

Similar to many previous studies [12-28], this study’s findings showed that the coaching session bolstered by tailored text messages led to significant within-group behavior change for the self-selected goal. These behavior changes were substantial in magnitude. The findings also suggest that there was a small ripple effect to stimulate improvement in exercise health behaviors even when exercise was not the self-selected behavior goal.

The importance of the improved exercise habits is underscored by the clinically relevant improvement in fasting glucose levels in the intervention group compared with the control group. These objective data were validating for the subjective self-reported exercise levels. Although the absolute decrease in serum glucose values appears to be modest (on average 4.6 mg/dL for the intervention participants), there is epidemiological evidence supporting the clinical relevance of this amount of glucose lowering when sustained over time. A recent prospective study in healthy individuals who did not have diabetes or cardiovascular disease showed that each 3 mg/dL increase in fasting glucose, even in the clinically accepted normal range, predicted a 1% increased risk of developing coronary disease over a mean of 4.3 years [36]. This finding supports the observation that even small improvements in health behaviors begun at a young age can produce major health improvements in the long term.

Researchers conducting numerous systematic reviews and meta-analyses analyzing text message–based studies have concluded that despite findings that text message interventions do work to encourage adherence to therapy, there are methodological issues with research in this area [13,15,18,25,37,38]. The current protocol aimed to address several of these issues. For example, the attributes of text messages such as content and dosage are often not described or evaluated to determine how those factors influence the results [18,25,37]. Additionally, text messages are sometimes standardized and generic for mass audiences or targeted for a specific health topic [38] (ie, smoking cessation, weight loss). It has been suggested that the tailoring of messages to a specific individual can enhance effect [17,39,40]. The current protocol provided a customized approach in which (1) participants could self-select an area of their health they elected to address and (2) the messages they received were tailored in response to preparatory work with focus groups and by feedback they sent to their coaches.

Another methodological design issue that the current protocol was designed to address was the use of objective data collection to evaluate outcome measures in tandem with subjective self-report progress toward their goal. To this end, fasting glucose values in the intervention group were significantly lower compared with the control group, in parallel with the increased exercise levels attained.

### Limitations

There are limitations to this study. One limitation in the study design is that the intervention comprised two components: (1) a single counseling session with goal setting and (2) an 8-week period of SMS text message support. It is therefore not possible to determine which of these components singly or together was responsible for the positive effects of the intervention. Limiting the intervention to one of these components would have enabled the investigators to report the effect of counseling or of text messaging. Future research on text messaging would benefit from unbundling such an intervention, perhaps by providing the counseling session to both the intervention and the control arms of the study. By setting the intervention to a single component such as text message support, it would be feasible to investigate the different effects of generic text messages versus texts with tailored, personalized feedback. Such an investigation is worthy of attention because generic texts can be automated for distribution, a much less time-consuming process than providing texts with personalized content.

The study did have statistical power to demonstrate a significant change in overall healthy behaviors in response to the intervention. However, the study may have been underpowered to find significant changes for the different individually selected behaviors with sample sizes of 6-10 participants. The small sample sizes likely explain why none of the analyses for separate behaviors were significant.

Another limitation of this report is the lack of analysis of the reports received by the health coaches for weekly behavioral compliance of the participants. This lack of analysis stemmed from the decision to specify *a priori* an overall 8-week outcome to the intervention. However, future studies may find this information helpful to interpret how well the individualized or customized the text messages worked. It may also be useful to interpret the overall burden of the intervention as experienced by the participants.

The study intervention lasted only 8 weeks, a trial period that was insufficient to capture significant improvements in some measures such as cholesterol changes. With this short study duration, it was also not possible to determine whether or not behavioral gains would be maintained over longer periods.

By design and in order to avoid confounding variables, the study excluded participation by students who had chronic illnesses or who took prescription medications other than for birth control. Furthermore, the enrolled population was predominantly white women. These factors do limit the ability to generalize the findings of this study to the student population at large.

### Conclusions

The findings from this SHUPEP study do support the current body of literature evaluating the utility and effectiveness of tailored mobile-based health communications as a successful strategy for improving health behaviors [17-39]. A new finding from this study suggests that there may be a mild ripple effect of this technological intervention on other health outcomes outside of the targeted behavior. Perhaps the main takeaway message for care providers on other college campuses is that the face-to-face feedback and behavioral health goal-setting along with timely motivational text messages as described in this report are of value in promoting healthy behaviors in college students. Care providers for college students should be emboldened to embrace digital technologies to augment their efforts for health promotion.

## Abbreviations

BA: behavioral assessments
ESS: Epworth Sleepiness Scale
FS: Visual Analog Fatigue Scale
IPAQ: International Physical Activity Questionnaire
MET: metabolic equivalent
PSQI: Pittsburgh Sleep Quality Index
PSS: Perceived Stress Scale
RYP: Rate Your Plate

## Appendix

Multimedia Appendix 1 CONSORT eHealth Checklist.
