# Supplementary material for: Effect of a Counseling Session Bolstered by Text Messaging on Self-Selected Health Behaviors in College Students: A Preliminary Randomized Controlled Trial
Source: JMIR Mhealth Uhealth. 2017 May 17;5(5):e67. doi: 10.2196/mhealth.6638 (PMC5451640; doi:10.2196/mhealth.6638)
Supplement: Multimedia Appendix 1 [file mhealth_v5i5e67_app1.pdf]

CONSORT eHealth Check List

TITLE & ABSTRACT

1a Identification as a randomized trial in the title

Effect of a Counseling Session Bolstered by Text Messaging on Self-Selected Health Behaviors in College Students: A Preliminary Randomized Controlled Trial

i) Identify the mode of delivery in the title. See above

ii) Mention non-web-based components or important co-interventions in the title, if any. See above

iii) Mention primary condition or target group in the title, if any. See above

1b Structured summary of trial design, methods, results, and conclusions

Methods (in Abstract):

i) Mention key features/functionalities/components of the intervention and comparator in the abstract. If possible, also mention theories and principles used for designing the site. Keep in mind the needs of systematic reviewers and indexers by including important synonyms.
Of 60 participants, 30 were randomized to receive a single face-to-face meeting with a health coach to review results of behavioral questionnaires and to set a health behavior goal for the 8-week study period. The face-to-face meeting was followed by SMS text messages designed to encourage achievement of the behavioral goal. A total of 30 control subjects underwent the same health and behavioral assessments at intake and program end but did not receive coaching or SMS text messages.

ii) Clarify the level of human involvement in the abstract. See above

iii) Open vs. closed, web-based (self-assessment) vs. face-to-face assessments in abstract: Full-time students aged 18-30 years were recruited by word of mouth and campus-wide advertisements (flyers, posters, mailings, university website). In this unblinded study, participants (intervention group and control group) completed validated questionnaires regarding health behaviors.

iv) Results in abstract must contain use data: The texting app showed that 87.31% (2187/2505) of messages were viewed by intervention participants. Furthermore, 28 of the 30 intervention participants and all 30 control participants provided outcome data.

v) Conclusions/Discussions in abstract for negative trials: Not a negative trial

INTRODUCTION

Background and objectives

2a Scientific background and explanation of rationale

i) Describe the problem and the type of system/solution that is object of the study: The college experience is often the first time when young adults live independently and make their own lifestyle choices. These choices affect dietary behaviors, exercise habits, techniques to deal with stress, and decisions on sleep time, all of which direct the trajectory of future health. There is a need for effective strategies that will encourage healthy lifestyle choices in young adults attending college.

ii) Scientific background, rationale: Because nearly all students have cell phones with them most of their waking time, text message–based interventions represent a promising opportunity to offer remote health coaching to students on the go. Since the introduction of mobile phone–based interventions to promote behavior change, numerous researchers have reported positive outcomes. For example, research has investigated the efficacy of mobile phone–based interventions on smoking cessation, weight loss, physical activity, and improving clinical outcomes of pregnancy [26-28]. A mobile phone–based intervention that encourages healthy changes in a variety of lifestyle behaviors would have clear advantages, offering efficiency as several health behaviors could be addressed through a single app. However, most mobile phone–based interventions have focused on a single health objective to date. This preliminary randomized controlled trial aimed to evaluate the effect of a single coaching session bolstered by tailored text messages on self-selected health goals in a sample of college students.

2b Specific objectives or hypotheses We hypothesized that the coaching plus text message–based intervention would result in students successfully attaining their specified goals in the intervention group, which was randomly selected from interested participants.

METHODS

Trial design

3a Description of trial design: Unblinded, randomized controlled trial with a one to one allocation ratio

3b Important changes to methods after trial commencement: No changes after commencement of trial

i) Bug fixes, Downtimes, Content Changes: No down times, no content changes

ii) other “unexpected events”: None

Participants

4a Eligibility criteria for participants

i) Computer / Internet literacy is often an implicit “de facto” eligibility criterion - this should be explicitly clarified: As the study population included college students aged 18 to 30 years, and the intervention utilized text messaging short message service (SMS), technical literacy was assumed.

ii) Open vs closed, web-based vs face-to-face assessments: Full-time students aged 18-30 years were recruited by word of mouth and campus-wide advertisements (flyers, posters, mailings, university website). In this unblinded study, participants (intervention group and control group) completed validated questionnaires regarding health behaviors and reviewed the questionnaire results in a face-to-face setting with health coaches.

4b Settings and locations where the data were collected

i) Clearly report if outcomes were (self-) assessed through online questionnaires (as common in web-based trials) or otherwise. See above

ii) Report how institutional affiliations are displayed to potential participants: All participants were full-time students at the university where the study was conducted. No other affiliations were advertised.

Interventions

5 The interventions for each group with sufficient details to allow replication, including how and when they were actually administered.

i) Mention names, credentials, affiliations of the developers, sponsors, and owners: Health coaches hired to perform the study were certified by the American Council on Exercise in health coaching. The health coaches disseminated intervention SMS text messages through a custom iOS application designed by programmers working with the Integrative Cardiac Health Project. Neither the developers of the App nor the authors of the study are owners of the App.

ii) Describe the history/development process of the application and previous formative evaluations (e.g., focus groups, usability testing), as these will have an impact on adoption/use rates and help with interpreting results. Before the creation of text message content, focus groups were conducted in order to understand preferences of the college student target population (A Roth, unpublished data, September, 2014). The dialogue captured during these focus groups suggested that college students preferred a mix of action-oriented, informational, and motivational messages that were succinct and sent no more than three times per week.

iii) Revisions and updating. Clearly mention the date and/or version number of the application/intervention. Participants in the intervention arm were asked weekly via the application to complete brief behavioral assessments (BA). The BA included a question addressing progress toward the self-selected goal. The coaches used BA

feedback to customize text messages to address barriers impeding goal attainment. Some participants asked for more motivational type of text messages, whereas other participants opted for action-oriented messages that instructed them on their behaviors. A third type of message preferred by some participants was an informational-type text. The coaches required approximately one hour for each intervention participant per week to read the brief BA feedback messages and to customize the text messages to be disseminated to each participant. The self-selected behavioral goal served as a basis for text messages designed to encourage goal attainment. Half of the messages sent as part of the intervention were directed toward the self-selected behavioral goal and half of the messages targeted other health behaviors more generally.

iv) Provide information on quality assurance methods to ensure accuracy and quality of information provided, if applicable. Supervision of the text message development was provided by the study's principal investigator.

v) Access: Describe how participants accessed the application, in what setting/context, if they had to pay (or were paid) or not, whether they had to be a member of specific group. Participants were not paid nor did they have to pay for access to the app. Study participants downloaded the iOS application to a university-issued iPad or a personal iPhone. Intervention messages were delivered on a regular schedule on Tuesdays and Thursdays at 9:00 AM and Saturdays at 11:00 AM for the study period of 8 weeks.

vi) Describe mode of delivery, features/functionalities/components of the intervention and comparator, and the theoretical framework See above.
vii) Describe use parameters: Intervention messages were delivered on a regular schedule on Tuesdays and Thursdays at 9:00 AM and Saturdays at 11:00 AM for the study period of 8 weeks. The schedule for delivery of messages was chosen to distribute the messages across the week at times that would not be disruptive of the students' living schedule. Students retained the freedom to check for messages at their convenience.

viii) Clarify the level of human involvement (care providers or health professionals, also technical assistance) in the e-intervention or as co-intervention. Certified health coaches met with participant in the intervention arm of the study to review behavioral health assessment questionnaires and to encourage selection of a single behavioral health goal for change during the 8 week intervention. Subsequently, the participants in the intervention arm received tailored text messages three times per week to encourage behavior changes as selected by the participants.

ix) Report any prompts/reminders used: See above regarding SMS.

x) Describe any co-interventions (incl. training/support): See above for careful description of co-interventions.

Outcomes

6a Completely defined pre-specified primary and secondary outcome measures, including how and when they were assessed

1) At baseline and following the 8-week intervention period, participants in both the intervention and control arms of the trial completed validated behavioral surveys assessing dietary habits, exercise activity, levels of perceived stress, and sleep practices. Blood testing for fasting glucose and cholesterol was also performed before and after the intervention. Because participants set unique behavioral goals, we used a standardized indicator that operationalized “success” as the percentage of each participant’s goal that was attained at the conclusion of the study. For example, at intake one participant completed 7758 steps per day. She set a goal of 10,000 steps per day, and by the completion of the study, she had completed 9008 steps per day. Thus, she attained 56% (ie, 100 × [9008−7758]/[10,000−7758]) of her goal. The intervention would be considered successful if the mean percentage of goal attainment was statistically significantly greater than 0.

ii) Describe whether and how “use” was defined/measured/monitored: The computer server was queried to compile information on messaging. During the course of the study, 1160 messages were sent to intervention participants and 982 messages (85%) were viewed.

iii) Describe whether, how, and when qualitative feedback was obtained from participants (e.g., through emails, feedback forms, interviews, focus groups). There was no analysis of the reports received by the health coaches for weekly behavioral compliance of the participants. This lack of analysis stemmed from the decision to specify a priori an overall 8-week outcome to the intervention.

6b Any changes to trial outcomes after the trial commenced: None

Sample size

7a How sample size was determined: The RYP dietary assessment tool served as an outcome measure to determine the sample size estimation because the investigators had a past experience with the RYP tool and could estimate an effect size of the intervention on this parameter. It was assumed that similar responsiveness in the other outcome measures would be seen and that by using a general “improvement score” for each behavioral dimension, participants could be offered the freedom to choose any of the four behaviors for concentration of their improvement efforts. Dietary changes in RYP were expected to average 10 (SD 2) points for the intervention group compared with zero change (same standard deviation) for the control group. With a power of 90% for rejecting the null hypothesis and using a one-sided test with alpha = 2.5% (as we expected an increase in dietary adherence and minimal chance of a decrease in adherence), the sample size was found to be 25 participants in each arm of the study. Accounting for a 30% dropout rate, a total of 70 participants would be recruited, 35 participants for each arm.

7b When applicable, explanation of any interim analyses and stopping guidelines: Not applicable.

Randomisation

8a Method used to generate the random allocation sequence NPT: The participants were randomly assigned in a one-to-one ratio by a computer program to one of two groups for the duration of the study.

8b Type of randomisation; No blocking was utilized.

Sequence generation

Allocation concealment mechanism

9 Mechanism used to implement the random allocation sequence In this unblinded trial participants were randomly assigned by a computer program without concealment of the allocation.

Implementation

10 Who generated the random allocation sequence, who enrolled participants, and who assigned participants to interventions: The Health Coaches hired to complete the study were responsible for determining the allocation sequence, participant enrollment and managing the intervention.

11 Blinding

i) No blinding occurred.

ii) As part of the informed consent procedure, participants were made aware that the study involved a flip-of-the-coin chance that they may experience different components of the study compared with another participant.

Statistical methods

12a Statistical methods used to compare groups for primary and secondary outcomes: Comparisons between participants randomized to the intervention group and those serving as controls were made using two-sample t tests or chi-square tests as appropriate, with a P value <.05 indicating statistical significance.

i) Imputation techniques to deal with attrition/missing values: For 2 participants in the intervention arm, end-of-study data were not obtained to determine success or failure of the intervention with respect to goal attainment. In order to maintain an intention-to-treat analysis, these 2 participants were assigned zero progress toward their behavioral goals and their data were retained in the final analysis.

12b Methods for additional analyses, such as subgroup analyses and adjusted analyses: No subgroup or adjusted analyses were performed.

Ethics & Informed Consent

X26

i) Comment on ethics committee approval: The university Institutional Review Board and the Human Research Protection Office of the US Army Medical Research and Materiel Command approved the study protocol for human subject research.

ii) Outline informed consent procedures: After face-to-face evaluation and determination of eligibility to participate in the trial, written informed consent was obtained from participants before enrollment.

iii) Safety and security procedures This was considered to be a minimal risk clinical trial though all clinical information on participants was password protected if electronically stored and lock and key protected if on hard copies.

RESULTS

Participant flow (a diagram is strongly recommended) See CONSORT flow diagram

13a For each group, the numbers of participants who were randomly assigned, received intended treatment, and were analyzed for the primary outcome: Of 60 enrolled participants, 30 were randomized to the intervention arm and 30 to the control arm. For 2 participants in the intervention arm (lost to follow-up), end-of-study data were not obtained to determine success or failure of the intervention with respect to goal attainment. In order to maintain an intention-to-treat analysis, these 2 participants were assigned zero progress toward their behavioral goals and their data were retained in the final analysis.

13b For each group, losses and exclusions after randomization, together with reasons: See CONSORT flow diagram and above paragraph.

Recruitment

14a Dates defining the periods of recruitment and follow-up Participants were recruited from January to May 2015.

14b Why the trial ended or was stopped early: Not applicable. Recruitment goals and participation were completed as planned.

Baseline data

15 A table showing baseline demographic and clinical characteristics for each group NPT: Table 2 outlines these data in detail.

Numbers analyzed

16 For each group, number of participants (denominator) included in each analysis and whether the analysis was by original assigned groups

i) Report multiple “denominators” and provide definitions: This has been addressed in the methods and results queries.

ii) Primary analysis should be intent-to-treat; secondary analyses could include comparing only “users”, with the appropriate caveats that this is no longer a randomized sample: See above.

Outcomes and estimation

17 For each primary and secondary outcome, results for each group, and the estimated effect size and its precision (such as 95% confidence interval)

i) Values for goal attainment were acceptable for a normal distribution (D_{27}=.95, P=.19; skewness=0.28, SE=0.45; and kurtosis=1.18, SE=0.87). Of the 30 participants who received the intervention, 22 (73 %) showed improvements in their self-declared behavioral goal, 6 of 30 (20%) did not make gains toward their behavioral goal, and 2 (7%) did not provide data that could be evaluated. For analysis, the 2 participants who failed to complete end-of-study assessments were assigned zero values for behavioral goal attainment and were therefore considered failures of the intervention. For the entire intervention group (n=30), the success rate of 88% was calculated by averaging the percentages of each participant’s goal (positive gains, negative, and zeros) that was attained at the conclusion of the study. The one-sample t test indicated that the percent of goal attainment of 88% (for n=30) was significantly greater than 0 (t_{26}=3.68, P<.001, Cohen d=0.71, 95% CI [38.56-136.11]). Our hypothesis that the coaching plus text intervention would help participants’ goal attainment was supported by the data.

ii) In addition to primary/secondary (clinical) outcomes, the presentation of process outcomes such as metrics of use and intensity of use (dose, exposure) and their operational definitions is critical. The computer server was queried to compile information on messaging. During the course of the study, 1160 messages were sent to intervention participants and 982 messages (85%) were viewed.

Ancillary analyses

18 Results of any other analyses performed, including subgroup analyses and adjusted analyses, distinguishing pre-specified from exploratory. Data were examined to determine whether or not the participants’ success depended upon the category of their self-selected behavioral goal. Of the 30 participants in the intervention group, 10 selected dietary goals, 8 selected exercise goals, whereas there were 6 participants each who selected goals in stress management and in sleep. Based upon the category of behavioral goal, there were no differences in means for percentage of goal attainment ($F_{3,20}$=.16, P=.92, partial eta-squared =

.29). The breakdown in the percentages of goal attainment across the 4 categories was diet (131%), exercise (75%), stress management (69%), and sleep (98%).

Harms

19 All important harms or unintended effects in each group

i) Include privacy breaches, technical problems. No harms or unintended effects occurred.

ii) Include qualitative feedback from participants or observations from staff/researchers. Qualitative feedback was not obtained from participants or research staff members.

Interpretation/ Principal Findings

22 Interpretation consistent with results, balancing benefits and harms, and considering other relevant evidence

i) Restate study questions and summarize the answers suggested by the data, starting with primary outcomes and process outcomes (use). The main research hypothesis of this study was that a single counseling session followed by a text message–based intervention would result in the attainment of a self-selected health behavior goal. A secondary analysis was performed to determine whether or not the intervention directed at a particular behavior might also have a broader effect by encouraging other healthful behavior changes outside of the behavior goal of choice. Similar to many previous studies, this study's findings showed that the coaching session bolstered by tailored text messages led to significant within-group behavior change for the self-selected goal. These behavior changes were substantial in magnitude. The findings also suggest that there was a small ripple effect to stimulate improvement in exercise health behaviors even when exercise was not the self-selected behavior goal.

ii) Highlight unanswered new questions, suggest future research. The intervention comprised to components: (1) a single counseling session with goal setting and (2) an 8-week period of SMS text message support. It is therefore not possible to determine which of these components singly or together was responsible for the positive effects of the intervention. Limiting the intervention to one of these components would have enabled the investigators to report the effect of counseling or of text messaging. Future research on text messaging would benefit from unbundling such an intervention, perhaps by providing the counseling session to both the intervention and the control arms of the study. By setting the intervention to a single component such as text message support, it would be feasible to investigate the different effects of generic text messages versus texts with tailored, personalized feedback. Such an investigation is worthy of attention because generic texts can be automated for distribution, a much less time-consuming process than providing texts with personalized content.

DISCUSSION

Limitations

20 Trial limitations, addressing sources of potential bias, imprecision, and, if relevant, multiplicity of analyses The study did have statistical power to demonstrate a significant change in overall healthy behaviors in response to the intervention. However, the study may have been underpowered to find significant changes for the different individually selected behaviors with sample sizes of 6-10 participants. The small sample sizes likely explain why none of the analyses for separate behaviors were significant. Another limitation of this report is the lack of analysis of the reports received by the health coaches for weekly behavioral compliance of the participants. This lack of analysis stemmed from the decision to specify *a priori* an overall 8-week outcome to the intervention. However, future studies may find this information helpful to interpret how well the individualized or customized the text messages worked. It may also be useful to interpret the overall burden of the intervention as experienced by the participants. The study intervention lasted only 8 weeks, a trial period that was insufficient to capture significant improvements in some measures such as cholesterol changes. With this short study duration, it was also not possible to determine whether or not behavioral gains would be maintained over longer periods.

Generalizability

21 Generalizability (external validity, applicability) of the trial findings: By design and in order to avoid confounding variables, the study excluded participation by students who had chronic illnesses or who took prescription medications other than for birth control. Furthermore, the enrolled population was predominantly white women. These factors do limit the ability to generalize the findings of this study to the student population at large.

OTHER INFORMATION

Registration 23

Registration number and name of trial registry: The study protocol was registered on ClinicalTrials.gov with trial number 375278-3 (ClinicalTrials.gov Identifier:
NCT02476604).

Protocol

24 Where the full trial protocol can be accessed, if available. ClincalTrials.gov

Funding

25 Sources of funding and other support, role of funders: This research was funded by the US Army Medical Research and Materiel Command by a grant to the Henry M Jackson Foundation for the Advancement of Military Medicine, Rockville,

Maryland, with Jackson Foundation Award Number: W81XWH-11-2-0178. The US Army Medical Research and Materiel Command and the Jackson Foundation for the Advancement of Military Medicine had no part in study design, data analysis, review, or approval of the manuscript.

Competing interests

X27 i) In addition to the usual declaration of interests (financial or otherwise), also state the “relation of the study team towards the system being evaluated”: The authors/evaluators are distinct from the developers/sponsors of the intervention.
